# Relationship between emotional intelligence, quality of life, and infertility stigma in infertile woman: A descriptive-correlational study

**DOI:** 10.18502/ijrm.v22i9.17479

**Published:** 2024-11-14

**Authors:** Mobina Anari, Mahboube Taebi, Mohammad Javad Tarrahi, Masoume Pirhadi

**Affiliations:** ^1^Department of Midwifery and Reproductive Health, School of Nursing and Midwifery, Isfahan University of Medical Sciences, Isfahan, Iran.; ^2^Department of Midwifery and Reproductive Health, School of Nursing and Midwifery, Reproductive Sciences and Sexual Health Research Center, Isfahan University of Medical Sciences, Isfahan, Iran.; ^3^Department of Epidemiology and Biostatistics, School of Health, Anesthesiology and Critical Care Research Center, Isfahan University of Medical Sciences, Isfahan, Iran.

**Keywords:** Infertility, Quality of life, Emotional intelligence.

## Abstract

**Background:**

Infertility is one of the major crises in couples' lives, affecting both mental and physical health. One of the most significant consequences of infertility is stigma; moreover, emotional intelligence and women's coping style with these consequences can affect the quality of this disorder.

**Objective:**

This study aimed to investigate emotional intelligence, infertility stigma, quality of life, and the relationship between these variables in infertile women.

**Materials and Methods:**

This descriptive-correlational study was conducted from April 2023 to February 2024 on 349 infertile women referred to “Hazrat Maryam Infertility Center in Shahid Beheshti hospital” and “Isfahan Fertility and Infertility Center” in Isfahan, Iran. Sampling was done using a convenience sampling method, and tools included 4 questionnaires, a demographic and midwifery information form, the quality of life questionnaire, the female-infertility-stigma-questionnaire, and the Schutte-Emotional-Intelligence scale.

**Results:**

The results showed that the women's mean age was 35.08 ± 7.60 yr. The mean score for emotional intelligence was 109.06 ± 6.12, and the infertility stigma's was 56.1 ± 14.73. The results indicated a positive correlation between emotional intelligence and infertility stigma (r = 0.13, p < 0.001). Only the mean score of social well-being and general health dimensions had a significant relationship with the total score of emotional intelligence (r = 0.037, p < 0.02).

**Conclusion:**

Infertile women have a lower quality of life and experience higher stigma. Since the quality of life and overall mental health affect the treatment process, also emotional intelligence plays an important role in human life, educational sessions and counseling should be considered in the treatment programs to improve the quality of life, thereby facilitating the treatment process with greater ease and speed.

## 1. Introduction

According to the World Health Organization definition, infertility means the inability to achieve clinical pregnancy after 12 months of regular and unprotected sexual intercourse. According to the latest World Health Organization report, infertility is a disease that causes disability in reproduction as a disorder of function (1). In Iran, in 2019, the overall prevalence of primary infertility among infertile women was 20.2%, indicating a high prevalence of primary infertility among Iranian women of childbearing age (2). A study in 2015 showed that the prevalence of infertility in Iran is 17.3% (3).

Studies have shown that infertility can affect various aspects of couples lives, causing negative effects on their quality of life, both psychologically and physically (4, 5). Infertile couples experience a high level of stress, such as pressure on marital, sexual, and social relationships, which significantly affects their quality of life (6). Another study has shown that infertile women have a lower quality of life than their fertile husbands (7). Additionally, the results of a comparative study that aimed to examine comprehensive infertility stigma among fertile and infertile women in the United States, showed that both infertile and fertile women believed that infertile women are stigmatized and that infertility stigma in infertile women is greater than in infertile men (8).

Infertile women in developing countries experience more negative consequences of childlessness compared to developed societies. Globally, childlessness creates problems for couples, especially for women, who are usually considered responsible for infertility and suffer from personal grief, social stigma, rejection, and serious economic deprivation (9). The majority of annoying feelings of infertility are associated with stigma. Stigma may reduce self-esteem and self-efficacy in these women and also could be accompanied with increased distress, downward social support, and weak social status (10). In this situation, individuals with high emotional intelligence can manage stress-inducing conditions and emotions arising from those conditions (11). According to the Bar-On model, emotional intelligence includes a set of noncognitive skills, abilities, and capacities that increase an individual's ability to cope with environmental pressures and demands effectively. Emotional intelligence is a set of abilities, skills, and capacities that individuals use for effective adaptation to life. Research has shown a significant relationship between emotional intelligence and quality of life, and emotional intelligence has even been considered a potential predictor of health in all populations (12, 13). Based on what has been stated, infertility is a stressful condition, and one of the most important consequences of infertility is stigma. On the other hand, women's coping style with this stress affects their quality of life, and their quality of life affects their adaptation to infertility.

The present study aimed to investigate emotional intelligence, infertility stigma, quality of life, and the relationship between these variables in infertile women.

## 2. Materials and Methods

This descriptive-correlational study was conducted on 349 infertile women attending infertility centers, “Hazrat Maryam Infertility Center in Shahid Beheshti hospital” and “Isfahan Fertility and Infertility Center” in Isfahan, Iran, and those who were selected using convenience sampling. The inclusion criteria included Iranian nationality, willingness to participate in the study, diagnosis of infertility by gynecologists and filing of cases in sampling centers, being aware of the cause of infertility, primary infertility based on the diagnosis recorded in the medical record, no history of previous pregnancy, and having minimum literacy in reading and writing to complete the questionnaires. The exclusion criterion was incomplete questionnaire completion.

### Sample size

The sample size was calculated considering the confidence level of 95%, alpha equal to 0.05 and beta 0.2, and the expected correlation coefficient of 0.15, according to the following formula, 347 people were considered, including the loss of 400 people.

The standard normal deviate for α = Z α = 1.96

The standard normal deviate for β = Z β = 0.84

C = 0.5 * ln [(1+r)/(1-r)] = 0.1511

Total sample size = N = [(Zα+Zβ)/C]^2^+3 = 347

After explaining the title and objectives of the study to the research units and obtaining their informed consent, questionnaires were provided to them for completion in an appropriate location while respecting their privacy. The tools used in this study included a demographic and midwifery information form, the quality of life questionnaire, the female infertility stigma questionnaire, and the Schutte Emotional Intelligence scale. The quality of life questionnaire comprised 36 questions and consisted of 8 subscales, each composed of 2–10 items. The 8 subscales of this questionnaire were “physical functioning, role limitations due to physical health, role limitations due to emotional problems, energy/fatigue, emotional well-being, social functioning, pain, and general health”. In this questionnaire, a lower score means a lower quality of life (14).

The women's infertility stigma questionnaire female infertility stigma instrument (ISI-F) consists of 20 items divided into 3 sections: “stigma profile" with 7 items, “self-stigma" with 6 items, and “escaping from stigma" with 7 items. The response scale to the questions is a 5-point Likert scale ranging from strongly agree to strongly disagree, with scores ranging from 1–5. Scores are reported between 20 and 100, with higher scores indicating a higher perception of infertility stigma.

The last version of ISI-F had 20 questions. Total content validity index and content validity ratio were 0.94 and 0.87. These subscales were “stigma profile (7 questions), self-stigma (6 questions), and escaping from stigma (7 questions)”. Internal consistency of the ISI-F have been approved by Cronbach's alpha (0.909) (15).

The Schutte Emotional Intelligence scale consists of 33 questions and aims to measure emotional intelligence components (emotion regulation, evaluation and expression of emotions, and emotional utilization) in individuals. The Schutte Emotional Intelligence scale was developed by Schutte et al., based on the initial model of Mayer and Salovey (16).

### Ethical Considerations

This study is registered at the Isfahan University of Medical Sciences, Isfahan, Iran and approved on March 11, 2023 (Code: IR.MUI.NUREMA.REC.1401.177). Before sampling, eligible people were informed about the objectives of the study and the confidentiality of the research team regarding their personal information was ensured and they were asked to sign the informed consent form.

### Statistical Analysis

The data were collected using the Statistical Package for the Social Sciences, version 21, SPSS Inc., Chicago, Illinois, USA and analyzed descriptively and inferentially. First descriptive analyses like frequency, mean, and standard deviation (SD) were done. Pearson correlation test was done to check the relationship among independent variables.

## 3. Results

The present study aimed to determine the relationship between emotional intelligence, infertility stigma, and quality of life in infertile women attending from April 2023 to February 2024 with 349 infertile women. The results of this study showed that the average age of women was 35.08 ± 7.60 yr and the average age of husbands was 28.8 ± 7.39 yr. Furthermore, the average duration of marriage was 9.70 ± 5.7 yr, and the average duration of infertility and infertility treatment was 61.8 ± 73.5 months and 51.03 ± 44.5 months, respectively. 73.6% of women were housewives, and the highest level of education among women was diploma (39%), followed by master's or higher (8%), associate or bachelor's degree (35%), junior high school (13.2%), and primary school (4.8%). Additionally, most women (44.1%) were somewhat satisfied with their economic conditions. The most commonly used types of treatment among these women were in vitro fertilization (66.5%), intrauterine insemination (14.9%), and intracytoplasmic sperm injection (4.6%), and 14% of them had used other infertility treatment methods. 92.8% of women did not mention any underlying diseases, but 2.9% of women (10 individuals) had diabetes, 2.6% (9 individuals) had kidney diseases, 1.1% (4 individuals) had cancer, and 0.6% (2 individuals) had cardiovascular diseases. In the following mean ± SD scores of emotional intelligence, quality of life, and infertility stigma in infertile woman is shown in table I.

The Pearson correlation test results showed a positive correlation between the total score of emotional intelligence and the total score of infertility stigma (p = 0.013). Furthermore, a significant correlation was found between the “emotion regulation" dimension and the total stigma score (p = 0.034).

Further data analysis showed a significant correlation between the mean score of the emotion regulation dimension of emotional intelligence and the mean scores of the “stigma profile" and “self-stigma" dimensions of infertility stigma (p = 0.023, p = 0.012). The findings indicated that among the dimensions of quality of life, only the mean scores of the emotional well-being and general health dimensions had a significant correlation with the total score of emotional intelligence (r = 0.11, p = 0.037, r = 0.12, p = 0.020). More information is shown in table II.

Based on the obtained results, it was observed that stigma profile (p = 0.002), self-stigma (p < 0.001), physical health (p = 0.021), mental health (p = 0.001), and total stigma (p = 0.002) in women who have employed and unemployed husbands had a statistically significant difference from each other. In women who had employed husbands, the amount of stigma profile, self-stigma, and total stigma were 2.5, 2.24, and 5.2 units were respectively less than in women who had unemployed husbands. In addition, the physical health and mental health of women with working husbands were 12.01 and 17.4 units higher than women with unemployed husbands. No statistical difference was observed between employed and unemployed women in the dimensions of emotion regulation, evaluation and expression of emotion, exploitation of emotion, stigma profile, self-stigma, escape from stigma, physical health, mental health, emotional intelligence, and total stigma.

Table III shows other research findings about the relationship between the characteristics of the studied subjects and the studied variables.

As shown in the table, there was a significant positive correlation between emotion regulation and duration of marriage. Also, this positive relationship was seen between exploitation of emotion and duration of infertility. It is interesting that there was a significant relationship between stigma profile and the age of the couple, but a significant negative correlation has been found between escape from stigma and man's age. Finally, it was shown to be significant positive correlation between total emotional intelligence and duration of marriage.

**Table 1:** Mean scores of emotional intelligence, quality of life, and infertility stigma in infertile woman

**Variables **		**Mean ± SD**	**Minimum**	**Maximum**
**Emotional intelligence**				
	**Excitement regulation**	34.78 ± 5.17	20	49
	**Evaluating and expressing emotions**	40.63 ± 5.39	21	56
	**Exploitation of excitement**	33.66 ± 4.83	19	47
	**Total emotional intelligence**	109.06 ± 12.04	66	145
**Quality of life scale**				
	**Physical performance**	85.62 ± 17.2	0	100
	**Role disorder due to physical health**	16.48 ± 27.28	0	100
	**Role disorder due to emotional health**	23.21 ± 34.45	0	100
	**Energy/fatigue**	65.92 ± 17.72	5	100
	**Emotional well-being**	68.86 ± 17.17	8	100
	**Social function**	75 ± 20.72	0	100
	**The pain**	81.37 ± 21.16	0	100
	**General health**	67.39 ± 17.54	8	100
	**Physical health**	250.85 ± 40.52	110.5	361
	**Mental health**	232.99 ± 46.13	94	345
**Infertility stigma**				
	**Stigma profile**	21.64 ± 6.78	7	35
	**Self-stigma**	14.72 ± 5.43	6	30
	**Escaping from stigma**	19.74 ± 5.74	7	35
	**Total stigma**	56.1 ± 14.73	66	145

**Table 2:** Pearson correlation coefficient between the total score and dimensions of emotional intelligence variables, quality of life, and infertility stigma

**Variables**	**Emotion regulation**	**Evaluation and expression of emotion**	**Exploitation of emotion**	**Total emotional intelligence**	**Physical health**	**Mental health**
**Stigma profile**	0.14*	0.19*	0.02	0.15*	-0.14*	-0.17*
**Self-stigma**	0.12*	0.21*	0.01	0.15*	-0.09	-0.18*
**Escaping from stigma**	0.02	0.1	-0.08	0.02	-0.1	-0.06
**Total stigma**	0.1*	0.2*	-0.02	0.13*	-0.14*	-0.17*
*It is significant at the 0.05 level						

**Table 3:** Pearson correlation coefficient between scales and sub-scales of variables with midwifery information

**Variables**	**Age of women**	**Age of husbands**	**Duration of infertility**	**Duration of treatment**	**Duration of marriage**
**Emotion regulation**	0.021	0.094	0.078	0.014	0.115*
**Evaluation and expression of emotion**	0.026	0.084	0.03	0.008	0.038
**Exploitation of emotion**	-36	-4	0.12*	0	0.162**
**Stigma profile**	0.121*	0.116*	-0.048	-0.092	-0.103
**Self-stigma**	-0.091	0.047	0.006	-0.024	-0.02
**Escaping from stigma**	-0.157**	-0.137*	-0.139**	-0.123**	-0.166**
**Physical health**	-0.044	-0.053	0.05	0.046	0.059
**Mental health**	-0.025	-0.069	0.07	0.066	0.035
**Total emotional intelligence**	0.006	0.076	0.095	0.01	0.131*
**Total stigma**	-0.15**	-0.124*	-0.074	-0.099	-0.119*
*It is significant at 0.05. **It is significant at the 0.01					

## 4. Discussion

This study shows that infertile women in developing countries experience more negative consequences of childlessness compared to developed societies. Childlessness, especially for women, leads to personal grief, social stigma, ostracism, and significant economic deprivation (9). In 2015, the posts and comments of 432 infertile American women were collected in the United States, who had inquired about treatment in online forums. It was found that these patients feel a strong stigma when talking about their infertility, either by themselves or by others, and therefore suffer from social isolation (17). The findings of our study as well as the mentioned studies show that in economically advanced countries and even in poor countries and the third world, there is a problem of suffering caused by infertility in women and the stigma of infertility, and this issue requires global determination to solve it. Most of the negative emotions associated with infertility are significantly associated with stigma. Stigma may harm self-esteem and empowerment in infertile women and is also associated with increased dissatisfaction, low social support, and poor social status (10).

The results of this study showed that the quality of life of infertile women was low; in this regard, the results of the study by Direkvand-Moghadam and colleagues, which was conducted to compare the quality of life in infertile women and women without infertility problems, demonstrated that the quality of life for an infertile women is significantly lower than for women without infertility issues. This study also showed that infertile women have worse conditions in the average scores of items related to physical and mental health and social functioning (18). Some studies indicate that 69.19% of infertile women experience stigma, and 53.8% also suffer from self-stigmatization (19, 20). Based on what was said, the decrease in the quality of life caused by infertility in women and the stigma of infertility makes women involved in a cycle of suffering, which may even reduce the physical and social health of these women more than expected by strengthening their negative feelings and their self-stigmatization.

Our study showed a positive correlation between total emotional intelligence scores and total infertility stigma scores, as well as a significant relationship between the “emotion regulation" dimension and total stigma score. This finding shows that in order to change the stigma felt in women, considering the difficulty of changing the thoughts and behavior of others, it is possible to invest in improving the emotional intelligence of infertile women so that more results can be achieved and better controlled.

Yokota, a Japanese researcher, stated that following an infertility diagnosis, infertile women experience guilt, shame, and self-blame due to fear of social and familial rejection. This leads to negative emotions, strained marital relationships, and reduced quality of life, affecting their normal lives (21). A study has shown that infertility, through both psychological and physical aspects, leads to a decrease in quality of life (22). Depression, anxiety, irrational beliefs such as perfectionism, sexual anxiety and stress, loss of self-confidence, feelings of guilt and shame, and sexual dysfunction resulting from infertility disorders, all contribute to a decrease in individuals quality of life (23). Although both spouses experience this crisis, investigations indicate that the negative psychological effects of infertility are greater for women compared to men, leading to a decrease in their quality of life (24). Additionally, the results of a comparative study examining comprehensive infertility distress among fertile and infertile women in the United States showed that both infertile and fertile women believe infertile women are stigmatized, and infertility distress is higher in infertile women than in infertile men (8). The results of this study showed that regardless of where people live, the stigma of infertility and its complications are more in women than in men. These findings are more understandable in Eastern cultures like our country.

In this study, employed women and unemployed women did not show statistical differences in terms of emotional intelligence factors and stigma dimensions, but in terms of the employment of their spouses, a statistically significant difference was observed. A study, aimed at investigating social distress among infertile women in China, showed that employed female patients and patients with higher incomes experienced less distress, which is inconsistent with the findings of this study. This discrepancy is likely due to differences in the research population and the cultural factors influences, as well as the differences in the instruments used. They used a Chinese version of the infertility stigma questionnaire with different questions and subscales (25). Correspondingly, an Iranian study aimed at determining effective factors on the quality of life of infertile women. The study revealed significant statistical correlations between quality of life and spouse's occupation, financial problems due to treatment, hope for treatment, intensity of desire for children, pressure from relatives to have children, and family economic status (26). It seems that the cultural differences in our society and the headship of men in Iranian families show the role of men's employment in the level of perceived stigma and the quality of life in women more than women's own employment.

Results indicated a significant correlation between emotional intelligence, total stigma, and quality of life with the age of the women, the age of their husbands, and the duration of their infertility. Various studies have shown that the duration of infertility diagnosis and treatment affects women's quality of life and perceived stigma. Specifically, as the duration of diagnosis and treatment increases, the quality of life deteriorates, and the level of perceived stigma increases. This may be due to hopelessness regarding successful treatment, economic pressures from infertility treatment, and social factors (27, 28). Additionally, Li and Zhu reported that the level of perceived stigma in infertile women who had been undergoing treatment for more than one year was higher than in those who had been undergoing treatment for less than one year (29). Due to potential pressure from their surroundings and public opinion, a significant number of infertile women decide to conceal their condition and even avoid seeking treatment, which delays their recovery and can disrupt their social health and ability to cope with individual and social problems (30).

The results of the present study showed that some dimensions of quality of life had a significant relationship with the total score of emotional intelligence. The findings of a study, which aimed to examine the relationship between emotional intelligence and quality of life in women with chronic diseases, indicated that emotional intelligence has a direct and significant relationship with quality of life (31). Since emotional intelligence represents how individuals behave in emotionally charged scenarios, it may act as a buffer against distress. Therefore, high emotional intelligence may be a protective factor against internalizing distress while also improving their quality of life.

## 5. Conclusion

The results of the present study indicated that infertile women have a lower quality of life and experience a higher level of stigma. Quality of life is one of the components that has a significant relationship with emotional intelligence. Given that the quality of life and mental health in general impact the treatment process, and since emotional intelligence is related to life satisfaction, the quality of interpersonal relationships, and job success (all of which play important roles in life) it is recommended to include educational and counseling sessions aimed at improving the quality of life, emotional intelligence, and perceived stigma in the treatment programs for infertile couples to facilitate and expedite the treatment process.

##  Data Availability

Data supporting the findings of this study are available upon reasonable request from the corresponding author.

##  Author Contributions

M. Anari: Concept and design, acquisition, analysis, or interpretation of data, drafting of the manuscript. M. Taebi: Concept and design. MJ. Tarrahi: Had full access to all of the data in the study and takes responsibility for the integrity of the data and the accuracy of the data analysis. M. Pirhadi: Concept and design, acquisition, analysis, or interpretation of data, drafting of the manuscript, supervision. Critical revision of the manuscript for important intellectual content: All authors.

##  Conflict of Interest 

The authors declare that there is no conflict of interest.
